# Supplementary material for: Comorbidity of age-related macular degeneration with Alzheimer’s disease: A histopathologic case-control study
Source: PLoS One. 2019 Sep 30;14(9):e0223199. doi: 10.1371/journal.pone.0223199 (PMC6768473; doi:10.1371/journal.pone.0223199)
Supplement: Attachment — Submitted filename: Response to Reviewers.docx [file pone.0223199.s001.docx]

PONE-D-19-17299
Comorbidity of age-related macular degeneration with Alzheimer’s disease: A histopathologic case-control study

We would like to thank the Academic Editor and the reviewers for taking the time to evaluate our manuscript and for their thoughtful, constructive comments. As requested, we have revised the manuscript. Below we detail our “point by point” responses to the reviews and we indicate the location of the modified material in the revised manuscript.

If you would like to make changes to your financial disclosure, please include your updated statement in your cover letter.

N/A

To enhance the reproducibility of your results, we recommend that if applicable you deposit your laboratory protocols in protocols.io, where a protocol can be assigned its own identifier (DOI) such that it can be cited independently in the future. For instructions see: [http://journals.plos.org/plosone/s/submission-guidelines#loc-laboratory-protocols](https://urldefense.proofpoint.com/v2/url?u=http-3A__journals.plos.org_plosone_s_submission-2Dguidelines-23loc-2Dlaboratory-2Dprotocols&d=DwMGaQ&c=imBPVzF25OnBgGmVOlcsiEgHoG1i6YHLR0Sj_gZ4adc&r=wr-8xl2HqgyJsLnwrKs9FtDGhMSOgDAA6DViv_eEE_o&m=ad6hD75BGtXRg29KyjwFwcG-71yVFKA3LHdpbEC0HjI&s=amN5UQGxePxVwxNFfYIT5TxCxg1Mwmu6E5npZntm3SI&e=)

N/A

1. When submitting your revision, we need you to address these additional requirements.

Please ensure that your manuscript meets PLOS ONE's style requirements, including those for file naming. The PLOS ONE style templates can be found at
[http://www.journals.plos.org/plosone/s/file?id=wjVg/PLOSOne_formatting_sample_main_body.pdf](https://urldefense.proofpoint.com/v2/url?u=http-3A__www.journals.plos.org_plosone_s_file-3Fid-3DwjVg_PLOSOne-5Fformatting-5Fsample-5Fmain-5Fbody.pdf&d=DwQGaQ&c=imBPVzF25OnBgGmVOlcsiEgHoG1i6YHLR0Sj_gZ4adc&r=wr-8xl2HqgyJsLnwrKs9FtDGhMSOgDAA6DViv_eEE_o&m=ad6hD75BGtXRg29KyjwFwcG-71yVFKA3LHdpbEC0HjI&s=tcevqGke87uLeb4VeLYUzqMqUIJLrNiwzyQ-4SCNMPk&e=) and [http://www.journals.plos.org/plosone/s/file?id=ba62/PLOSOne_formatting_sample_title_authors_affiliations.pdf](https://urldefense.proofpoint.com/v2/url?u=http-3A__www.journals.plos.org_plosone_s_file-3Fid-3Dba62_PLOSOne-5Fformatting-5Fsample-5Ftitle-5Fauthors-5Faffiliations.pdf&d=DwQGaQ&c=imBPVzF25OnBgGmVOlcsiEgHoG1i6YHLR0Sj_gZ4adc&r=wr-8xl2HqgyJsLnwrKs9FtDGhMSOgDAA6DViv_eEE_o&m=ad6hD75BGtXRg29KyjwFwcG-71yVFKA3LHdpbEC0HjI&s=Qa_86lzwdAKp5_nIGokcuLfoiK8zDv_py6F2DQtT-3E&e=)

2. We would be grateful if you could clarify whether the information included in the Supporting Information has been published previously, and if so, whether you have permission of the original copyright holder to reproduce these tables and images. If this information has been published previously we ask that you remove this information and include a relevant citation to the previous publications.

The tables included in the Supporting Information contain content that was previously published in the manuscripts referenced under 23-25. These references are cited appropriately in the Methods section (lines 115-120). The tables in the Supporting Information section were removed.

3. Please provide additional details regarding participant consent. Please state whether this was obtained from patients before death and/or from the next of kin. In the Methods section, please ensure that you have specified (1) whether consent was informed and (2) what type you obtained (for instance, written or verbal). If the need for consent was waived by the ethics committee, please include this information. Please also explain why the requirement for consent was waived.

The need for participant consent was waived by the Duke IRB for this decedent research, as all research subject are deceased, and all personal health information (PHI) was used solely for research and was not disclosed to anyone outside the Duke University without removing all identifiers.

This information was added to the Methods section (lines 135-139).

4. We note that Figure 1 in your submission contain copyrighted images. All PLOS content is published under the Creative Commons Attribution License (CC BY 4.0), which means that the manuscript, images, and Supporting Information files will be freely available online, and any third party is permitted to access, download, copy, distribute, and use these materials in any way, even commercially, with proper attribution. For more information, see our copyright guidelines: [http://journals.plos.org/plosone/s/licenses-and-copyright](https://urldefense.proofpoint.com/v2/url?u=http-3A__journals.plos.org_plosone_s_licenses-2Dand-2Dcopyright&d=DwQGaQ&c=imBPVzF25OnBgGmVOlcsiEgHoG1i6YHLR0Sj_gZ4adc&r=wr-8xl2HqgyJsLnwrKs9FtDGhMSOgDAA6DViv_eEE_o&m=ad6hD75BGtXRg29KyjwFwcG-71yVFKA3LHdpbEC0HjI&s=_z7rM9izFOi--dU8o2kdbh2BS5zezlcCQZwuKeDfdoU&e=).

We require you to either (1) present written permission from the copyright holder to publish these figures specifically under the CC BY 4.0 license, or (2) remove the figures from your submission:

1.    You may seek permission from the original copyright holder of Figure 1 to publish the content specifically under the CC BY 4.0 license.

We recommend that you contact the original copyright holder with the Content Permission Form ([http://journals.plos.org/plosone/s/file?id=7c09/content-permission-form.pdf](https://urldefense.proofpoint.com/v2/url?u=http-3A__journals.plos.org_plosone_s_file-3Fid-3D7c09_content-2Dpermission-2Dform.pdf&d=DwQGaQ&c=imBPVzF25OnBgGmVOlcsiEgHoG1i6YHLR0Sj_gZ4adc&r=wr-8xl2HqgyJsLnwrKs9FtDGhMSOgDAA6DViv_eEE_o&m=ad6hD75BGtXRg29KyjwFwcG-71yVFKA3LHdpbEC0HjI&s=FPMajmfPkpwt09D4ug4UwevVMoHUu7znlJdTe8eKZA0&e=)) and the following text:

“I request permission for the open-access journal PLOS ONE to publish XXX under the Creative Commons Attribution License (CCAL) CC BY 4.0 ([http://creativecommons.org/licenses/by/4.0/](https://urldefense.proofpoint.com/v2/url?u=http-3A__creativecommons.org_licenses_by_4.0_&d=DwQGaQ&c=imBPVzF25OnBgGmVOlcsiEgHoG1i6YHLR0Sj_gZ4adc&r=wr-8xl2HqgyJsLnwrKs9FtDGhMSOgDAA6DViv_eEE_o&m=ad6hD75BGtXRg29KyjwFwcG-71yVFKA3LHdpbEC0HjI&s=x4Q-PvpEIsSc8m6NbWubcXC9CiUwWxTn-g5xd_EoFBc&e=)). Please be aware that this license allows unrestricted use and distribution, even commercially, by third parties. Please reply and provide explicit written permission to publish XXX under a CC BY license and complete the attached form.”

Please upload the completed Content Permission Form or other proof of granted permissions as an "Other" file with your submission.

We uploaded, as an “Other” file the rights license document granting permission to publish Figure 1 and the email confirmation from the copyright holder, SpringerNature. The copyright holder noted that, per legal department advice, SpringerNature is unable to sign any outside forms such as the Content Permission Form. However, the email communication states that we are able to reuse the figure in an open access publication under the Creative Commons Attribution License (CCAL) CC BY 4.0 with appropriate reference to the source in the figure legend.

In the figure caption of the copyrighted figure, please include the following text: “Reprinted from [ref] under a CC BY license, with permission from [name of publisher], original copyright [original copyright year].”

This text was included under lines 151-2.

2.    If you are unable to obtain permission from the original copyright holder to publish these figures under the CC BY 4.0 license or if the copyright holder’s requirements are incompatible with the CC BY 4.0 license, please either i) remove the figure or ii) supply a replacement figure that complies with the CC BY 4.0 license. Please check copyright information on all replacement figures and update the figure caption with source information. If applicable, please specify in the figure caption text when a figure is similar but not identical to the original image and is therefore for illustrative purposes only.

N/A

5. Please include captions for your Supporting Information files at the end of your manuscript, and update any in-text citations to match accordingly. Please see our Supporting Information guidelines for more information: [http://journals.plos.org/plosone/s/supporting-information](https://urldefense.proofpoint.com/v2/url?u=http-3A__journals.plos.org_plosone_s_supporting-2Dinformation&d=DwQGaQ&c=imBPVzF25OnBgGmVOlcsiEgHoG1i6YHLR0Sj_gZ4adc&r=wr-8xl2HqgyJsLnwrKs9FtDGhMSOgDAA6DViv_eEE_o&m=ad6hD75BGtXRg29KyjwFwcG-71yVFKA3LHdpbEC0HjI&s=Ok9V7GWMI9FZO9uMHx9hBvHA5eeyh6UY6vM4A13KqT4&e=).

 N/A. The Supporting Information was removed, per # 2 above.

Additional Editor Comments:

This manuscript presented a retrospective pathology-based case-control study which investigates whether there is association between Alzheimer's disease (AD) and age-related macular degeneration (AMD). The study did not find a statistically significant association between AD and AMD and suggested that any shared characteristics between the two disease may be nondeterministic. This work received favorable review from expert reviewers and minor revision is recommended to address the issues raised by the reviewers.


Reviewers' comments:

Reviewer's Responses to Questions

**Comments to the Author**

1. Is the manuscript technically sound, and do the data support the conclusions?

The manuscript must describe a technically sound piece of scientific research with data that supports the conclusions. Experiments must have been conducted rigorously, with appropriate controls, replication, and sample sizes. The conclusions must be drawn appropriately based on the data presented. 

Reviewer #1: Partly

Reviewer #2: Yes

2. Has the statistical analysis been performed appropriately and rigorously? 

Reviewer #1: I Don't Know

Reviewer #2: Yes

3. Have the authors made all data underlying the findings in their manuscript fully available?

The [PLOS Data policy](https://urldefense.proofpoint.com/v2/url?u=http-3A__www.plosone.org_static_policies.action-23sharing&d=DwMGaQ&c=imBPVzF25OnBgGmVOlcsiEgHoG1i6YHLR0Sj_gZ4adc&r=wr-8xl2HqgyJsLnwrKs9FtDGhMSOgDAA6DViv_eEE_o&m=ad6hD75BGtXRg29KyjwFwcG-71yVFKA3LHdpbEC0HjI&s=8DqPxd4KkxY8YEd2yxoImWI08fT305bUDQQ8mH0J7sE&e=) requires authors to make all data underlying the findings described in their manuscript fully available without restriction, with rare exception (please refer to the Data Availability Statement in the manuscript PDF file). The data should be provided as part of the manuscript or its supporting information, or deposited to a public repository. For example, in addition to summary statistics, the data points behind means, medians and variance measures should be available. If there are restrictions on publicly sharing data—e.g. participant privacy or use of data from a third party—those must be specified.

Reviewer #1: Yes

Reviewer #2: Yes

4. Is the manuscript presented in an intelligible fashion and written in standard English?

PLOS ONE does not copyedit accepted manuscripts, so the language in submitted articles must be clear, correct, and unambiguous. Any typographical or grammatical errors should be corrected at revision, so please note any specific errors here.

Reviewer #1: Yes

Reviewer #2: Yes

5. Review Comments to the Author

Please use the space provided to explain your answers to the questions above. You may also include additional comments for the author, including concerns about dual publication, research ethics, or publication ethics. (Please upload your review as an attachment if it exceeds 20,000 characters)

Reviewer #1: This is study on a very important question in the field of neurodegeneration both for AMD and for Alzheimer.
The study is rather well done and the literature review and context placement is very well done. The authors note the limitations very well which are unavoidable in a study for this nature that requires brain and eye autopsy specimens. Thus despite the limitations this study adds significantly to our knowledge.
One additional control that may be added is that of a the AMD prevalence in a cohort of controls patients form the electronic data record with the same demographics as the AD autopsy group to cover for the imbalances noted by the authors in their control group. Of course this will have its own limitations but it will be nice to know this information if possible.
Thank you

We would like to thank the reviewer for the very supportive comments.

In order to attempt to fulfill this request, we completed a Duke Enterprise Data Unified Content Explorer (DEDUCE) search of the electronic data records from all normal, control patients seen at the Duke Medical Center over the past 37 years with the same demographics as the AD autopsy group (aged 75 and above). We found a number of 486,395 patients. Among these subjects, only 12,140 also carried the diagnosis of AMD, consistent with a prevalence of 2.49%, which is much lower and not consistent with estimates from prior studies (references 1, 27). This reflects the fact that only a minority of patients were seen at Duke Eye Center for a dilated examination that would allow a diagnosis of AMD to be made. A true prevalence of AMD in this population would only be possible if only the patients that received both a dilated eye examinations at Duke and a neurocognitive evaluation to evaluate AD were considered in this analysis. Unfortunately, these numbers are not available to us at this time. The reviewer’s astute suggestion was added to the Discussion section as an important future direction (lines 372-375):

“To control for the imbalances in demographics and comorbidities noted between the AD and non-AD group, a future direction would be the calculation of the AMD prevalence from the electronic data in a cohort of control patients record with the same demographics as the AD group.”

Reviewer #2: This paper is a clearly written description of a methodologically rigorous study on an important topic. As the authors outline, the use of often elusive histopathological diagnoses makes this paper an impactful contribution to the field. It contains sufficient statistical information in general (though I wonder why the test statistic was given for chi-squared tests but not for t-tests), and thoughtful reflection in the discussion.

We thank the reviewer for the positive and constructive feedback.

The point on the test statistic is well taken, and for completeness, we are now reporting the test statistic for t-tests and z-tests in addition to chi-squared tests.

I commend this write up as an excellent example of being as close to publication-ready as a submission can be. Well done!
- On page 15, line 310, change "Alike..." to "Like..." or similar.

“Alike” was replaced with “Similar to”
- Some figures - if cost and space allow, which they may not - of examples of the histopathological findings would be engaging. Not essential.

While a figure showcasing histopathological examples of stages of AMD and AD would be indeed engaging, this is not feasible due to the many panels necessary and current space limitations. However, we believe that the diagram in Figure 1 concisely illustrates the Sarks stages of AMD, and the definitions of Braak and Braak stages are included in the Methods section.

- In the section, at the end of pages 14 and beginning of page 15, on the lack of shared polymorphisms between AMD and AD, consider citing this: Am J Geriatr Psychiatry. 2015 Dec;23(12):1290-1296. doi: 10.1016/j.jagp.2015.06.005 Its of direct relevance but my knowledge of this paper is because I am one of the authors, so clearly have an interest to declare on this point! The authors may well be aware of this paper and have decided against citing it, which is obviously fine. I also declare that I am the first author cited in reference 10.

We appreciate this suggestion and have now cited this informative reference.
